# Supplementary material for: Presence of Zonula Occludens Toxin-Coding Genes among Vibrio parahaemolyticus Isolates of Clinical and Environmental Origin
Source: Microorganisms. 2024 Feb 29;12(3):504. doi: 10.3390/microorganisms12030504 (PMC10974384; doi:10.3390/microorganisms12030504)
Supplement: Supplementary file 1 [file microorganisms-12-00504-s001.zip › microorganisms-2877305-supplementary.pdf]

Supplementary materials

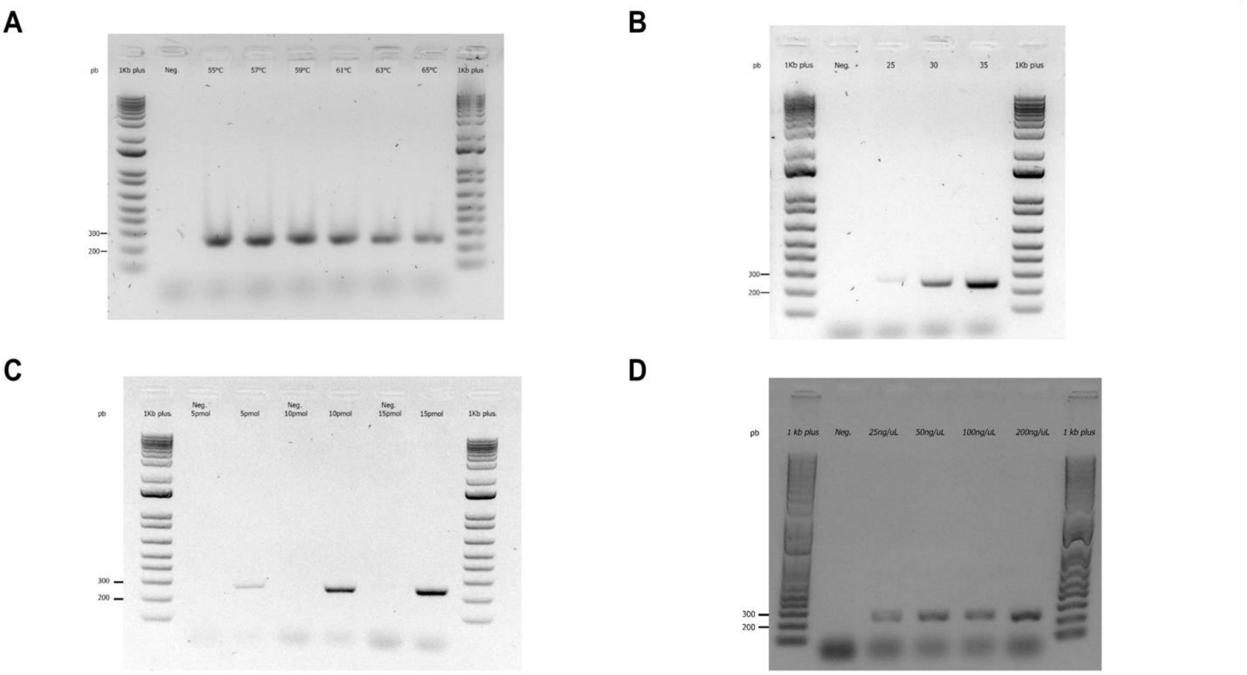

Figure S1. Standardization and validation of PCR assays for *V. parahaemolyticus zot* (A) Alignment temperature of *zot* primers for *V. parahaemolyticus.* Each lane shows an amplification result for each temperature analyzed, Neg: negative control without template DNA. (B) Amplification cycles for detection of *zot* in *V. parahaemolyticus*. Each lane shows the amplification result for cycles 25, 30 and 35, Neg: negative control without template DNA. (C) Amount of primer for the detection of *zot* in *V. parahaemolyticus.* Each lane shows the amplification result for 5, 10 and 15 pmol of each primer, Neg: negative control without template DNA. (D) Amount of gDNA for the detection of *zot* in *V. parahaemolyticus*. Each lane shows the amplification result for the quantities analyzed, Neg: negative control without template DNA. Image of the 1% w/v agarose gel. The molecular size standard corresponds to Ladder 1kb plus and the size of the bands that allow the identification of the resulting amplicon is indicated.

Table S1. Environmental isolates of *V. parahaemolyticus* from bivalve shellfish harvested in the Northern Adriatic Sea (Italy).

ID	Date	Geographic origin	Source	*toxR*	*tdh*	*trh*	TTSS1	TTSS2a	TTSS2b	*zot*
**1122**	30/05/2012	Northern Adriatic Sea	clams	1	0	1	1	0	1	0
**1128**	13/06/2012	Northern Adriatic Sea	clams	1	0	0	1	0	0	0
**1129**	19/06/2012	Northern Adriatic Sea	clams	1	0	1	1	0	1	0
**1140**	28/08/2012	Northern Adriatic Sea	clams	1	0	0	1	0	0	1
**1141**	29/08/2012	Northern Adriatic Sea	clams	1	0	0	1	0	0	0
**1147**	26/09/2012	Northern Adriatic Sea	clams	1	0	0	1	0	0	0
**1158**	24/10/2012	Northern Adriatic Sea	clams	1	0	1	1	0	1	0

**1162**	14/11/2012	Northern Adriatic Sea	clams	1	0	0	1	0	0	0
**1163**	15/11/2012	Northern Adriatic Sea	clams	1	0	0	1	0	0	0
**1164**	21/11/2012	Northern Adriatic Sea	clams	1	0	1	1	0	1	0
**1172**	13/12/2012	Northern Adriatic Sea	clams	1	0	0	1	0	0	1
**1182**	14/05/2013	Northern Adriatic Sea	mussels	1	0	0	1	0	0	1
**1204**	19/11/2013	Northern Adriatic Sea	clams	1	0	0	1	0	0	1
**1213**	03/03/2014	Northern Adriatic Sea	clams	1	0	0	1	0	0	0
**1214**	06/03/2014	Northern Adriatic Sea	clams	1	0	0	1	0	0	1
**1215**	12/03/2014	Northern Adriatic Sea	clams	1	0	1	1	0	1	0
**1219**	06/05/2014	Northern Adriatic Sea	mussels	1	0	0	1	1	0	0
**1220**	09/05/2014	Northern Adriatic Sea	clams	1	0	0	1	1	0	0
**1221**	09/05/2014	Northern Adriatic Sea	clams	1	0	1	1	0	1	1
**1225**	29/05/2014	Northern Adriatic Sea	clams	1	0	1	1	0	1	0
**1226**	30/05/2014	Northern Adriatic Sea	clams	1	0	1	1	0	1	0
**1231**	18/06/2014	Northern Adriatic Sea	clams	1	0	0	1	0	0	0
**1234**	30/06/2014	Northern Adriatic Sea	clams	1	0	1	1	0	1	0
**1237**	01/07/2014	Northern Adriatic Sea	clams	1	0	0	1	0	0	1
**1242**	04/08/2014	Northern Adriatic Sea	clams	1	0	0	1	0	0	1
**1246**	21/08/2014	Northern Adriatic Sea	clams	1	0	0	1	0	0	0
**1252**	02/09/2014	Northern Adriatic Sea	clams	1	0	0	1	0	0	0
**1260**	06/10/2014	Northern Adriatic Sea	clams	1	0	0	1	0	0	0
**1262**	09/10/2014	Northern Adriatic Sea	clams	1	0	0	1	0	0	1
**1269**	10/11/2014	Northern Adriatic Sea	clams	1	0	0	1	0	0	1
**1272**	10/11/2014	Northern Adriatic Sea	clams	1	0	0	1	0	0	0
**1275**	18/11/2014	Northern Adriatic Sea	clams	1	0	0	1	0	0	0

**1282**	02/12/2014	Northern Adriatic Sea	clams	1	0	0	1	0	0	0
**1291**	21/04/2015	Northern Adriatic Sea	oysters	1	0	0	1	0	0	1
**1292**	27/04/2015	Northern Adriatic Sea	clams	1	0	0	1	0	0	0
**1300**	06/07/2015	Northern Adriatic Sea	clams	1	0	0	1	0	0	0
**1303**	08/07/2015	Northern Adriatic Sea	clams	1	0	0	1	0	0	0
**1304**	16/07/2015	Northern Adriatic Sea	clams	1	0	0	1	0	0	0
**1326**	14/12/2015	Northern Adriatic Sea	mussels	1	0	0	1	0	0	0
**1329**	21/12/2015	Northern Adriatic Sea	clams	1	0	0	1	0	0	0
**1332**	26/01/2016	Northern Adriatic Sea	clams	1	0	0	1	0	0	0
**1340**	16/05/2016	Northern Adriatic Sea	clams	1	0	1	1	0	1	0
**1341**	18/05/2016	Northern Adriatic Sea	clams	1	0	0	1	1	0	0
**1344**	24/05/2016	Northern Adriatic Sea	mussels	1	0	0	1	0	0	0
**1346**	25/05/2016	Northern Adriatic Sea	mussels	1	0	0	1	0	0	0
**1349**	06/06/2016	Northern Adriatic Sea	clams	1	0	0	1	1	0	0
**1354**	13/06/2016	Northern Adriatic Sea	clams	1	0	0	1	0	0	1
**1372**	07/07/2016	Northern Adriatic Sea	clams	1	0	0	1	0	0	0
**1395**	10/08/2016	Northern Adriatic Sea	clams	1	0	0	1	0	0	1
**1404**	20/09/2016	Northern Adriatic Sea	mussels	1	0	0	1	0	0	0
**1411**	06/10/2016	Northern Adriatic Sea	clams	1	0	0	1	0	0	1
**1419**	25/10/2016	Northern Adriatic Sea	clams	1	0	0	1	0	0	0
**1425**	07/11/2016	Northern Adriatic Sea	clams	1	0	0	1	0	0	0
**1429**	09/11/2016	Northern Adriatic Sea	mussels	1	0	0	1	0	0	0

1: presence and 0: absence.
